# Evidence Supporting Utility of the Oral Cholate Challenge Test of Liver Function and Physiology to Aid Clinical Management: A Review of Analytical and Clinical Validation Studies

**DOI:** 10.1016/j.gastha.2026.100993

**Published:** 2026-05-08

**Authors:** Andrew P. Keaveny, Kamran Qureshi, Mitchell Shiffman, Timothy R. Morgan, K. Rajender Reddy, Joanne C. Imperial, Michael P. McRae, Touraj Shokati, Gregory T. Everson

**Affiliations:** 1 Division Gastroenterology and Hepatology, Mayo Clinic, Jacksonville, Florida; 2 Division of Gastroenterology and Hepatology, Saint Louis University School of Medicine, St. Louis, Missouri; 3 Bon Secours Liver Institute of Virginia, Richmond, Virginia; 4 Division of Gastroenterology, University of California, Irvine, Irvine, California; 5 Division of Gastroenterology, University of Pennsylvania Perelman School of Medicine, Philadelphia, Pennsylvania; 6 HepQuant, LLC, Denver, Colorado; 7 Custom DX Solutions LLC, Houston, Texas

**Keywords:** Cholate, Liver Function, Portal Hypertension, Varices, Clinical Outcomes, Clinical Trials

## Abstract

Current standard liver assessments rely on static laboratory values that reflect advanced disease or liver injury rather than overall hepatic health. This limitation highlights the need for dynamic measures of liver function to support accurate risk stratification, therapeutic monitoring, and clinical trial design. Quantitative liver function tests address this gap by providing physiologically based measures of hepatic clearance. Among these, cholate clearance is uniquely suited to assess hepatocellular function and portal-systemic shunting—key determinants of disease severity and prognosis. The oral cholate challenge test (OCCT), HepQuant DuO, is a simple, noninvasive method for quantifying cholate clearance using stable isotope-labeled cholate and liquid chromatography–tandem mass spectrometry analysis. This review highlights the test’s analytical and clinical validation across the spectrum of chronic liver disease. The test’s output parameters, Disease Severity Index, SHUNT%, and hepatic reserve, correlate with portal hypertension, varices, and risk for clinical outcomes. Applications for guiding endoscopic screening, predicting decompensation, and monitoring treatment response are highlighted. Comparative analyses show that the OCCT parameters outperform conventional laboratory tests of liver function and may complement anatomic or physical measurements, such as histology, magnetic resonance imaging, or elastography. Additional data supports its role as a sensitive biomarker and reasonably likely surrogate endpoint for drug trials of chronic liver disease. The OCCT addresses critical gaps in chronic liver disease and drug development and has the potential to transform the assessment of the patient with chronic liver disease.

## Introduction

Conventional liver tests reflect injury or advanced disease rather than true functional capacity, limiting their ability to guide risk stratification or therapeutic decisions. Quantitative liver function tests address this gap by measuring liver-specific uptake and perfusion, with cholate clearance uniquely suited to assess hepatocellular function and portal-systemic shunting. The oral cholate challenge test (OCCT) provides a simple, noninvasive measure of cholate clearance using stable isotope labeling and liquid chromatography–tandem mass spectrometry, enabling functional characterization across the spectrum of chronic liver disease. This review summarizes analytical and clinical validation of the OCCT and its applications in evaluating the portal circulation, predicting clinical outcomes, and monitoring treatment effects.

## Principles of Quantitative Testing of Liver Function

Quantitative tests of liver function use liver-specific substrates that are cleared from blood by hepatic uptake, metabolism, or both.^1^, ^2^, ^3^ In contrast to routine laboratory tests, which generally only reveal substantial loss of hepatic function once decompensation has occurred, quantitative clearance tests can detect subtle impairments in liver function earlier in the disease process. High-extraction substrates (>70% first-pass extraction and short elimination half-lives) depend on liver blood flow for clearance and can therefore be used to estimate hepatic perfusion.^4^, ^5^, ^6^, ^7^, ^8^, ^9^ Low-extraction substrates (<30% first-pass extraction and long elimination half-lives) reflect hepatic metabolic capacity rather than perfusion.^10^, ^11^, ^12^, ^13^, ^14^, ^15^, ^16^, ^17^, ^18^, ^19^, ^20^, ^21^

Metabolism of a substrate may also release volatile gas, such as CO_2_, which can be measured in exhaled breath. With 13C breath tests, a 13C label is synthetically introduced at the substrate's site of enzyme activity so that the rate of production of 13CO_2_ is related to the amount and activity of enzyme.^16^ Variations in the kinetics of CO_2_ between studies may be related, in part, to the route of administration of the test dose.^17^^,^^22^

The best-studied breath test of hepatic function that has been related to clinical outcomes is the 13C-methacetin breath test. Recent publications of generally small studies have attested to the ability of this test to predict mortality in patients with hepatocellular carcinoma (HCC) undergoing transarterial chemoembolization,^23^ the likelihood of recovery from acute liver failure,^24^ and risk for clinically significant portal hypertension (CSPH).^25^ However, the test may be affected by liver fat,^26^^,^^27^ transporter polymorphisms,^28^ and concomitant administration of over-the-counter products and medications.

Other breath tests using stable isotopically labeled α-ketoisocaproic acid, methionine, octanoic acid, and phenylalanine have shown promise at uncovering not only hepatic but also systemic impairment, especially in metabolic dysfunction–associated steatohepatitis (MASH).^29^ Limonene breath tests may correlate with cirrhosis and may predict risk for PH.^25^ Others have combined substrates (isoprene, limonene, and dimethyl sulfoxide) into a battery of breath analyses and demonstrated the ability to provide a holistic mapping of liver function.^30^

Gadoxetic acid is transported by hepatocytes in a fashion similar to indocyanine green. Its liver uptake and transport in bile can be tracked by magnetic resonance imaging, and these kinetic parameters may outperform indocyanine green in predicting post-hepatectomy liver failure.^31^ In addition, this technique has shown promise in assessing severity of hepatocellular impairment in clinical trials of drug therapy of MASH.

## The Oral Cholate Challenge Test

Cholate clearance is hepatocyte-specific, flow-dependent, and correlates with functional acinar mass^8^^,^^32^, ^33^, ^34^, ^35^; however, endogenous cholate is unsuitable as an estimate for clearance given its fluctuations in peripheral blood concentration due to enterohepatic cycling. The solution is to administer a distinguishable form of cholate and directly measure its clearance.

**Figure 1:**
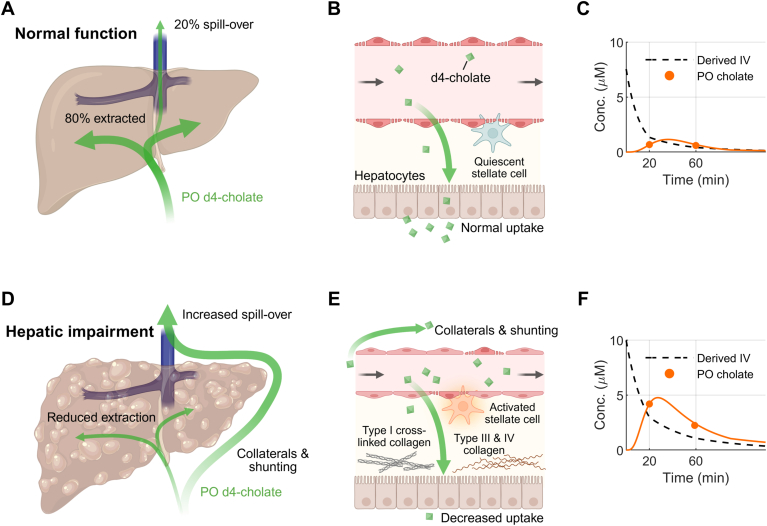
Overview of the oral cholate challenge test (HepQuant DuO). Oral d4-cholate is delivered to the liver via the portal vein. In health (A), 80% is removed by hepatic uptake in a first pass through the liver, and ∼20% (SHUNT%) spills over into the systemic circulation (B), and the measured area under the clearance curve for d4-cholate in peripheral blood is relatively low (C). With disease (D), diminished hepatocyte uptake and collateral circulation increase the spillover of the d4-cholate into the systemic circulation (E), and the area under the clearance curve increases (F). Created in BioRender. McRae, M. (2026) https://BioRender.com/91jtlui.

The OCCT (HepQuant DuO) is a simple, noninvasive method for quantifying the liver-specific uptake of cholate. In performing the OCCT, deuterium-labeled cholate (d4-cholate) is administered orally, and 2 blood samples are obtained at 20 and 60 minutes. Serum concentrations of d4-cholate are measured by liquid chromatography–tandem mass spectrometry.^36^^,^^37^ Since cholate’s uptake is flow-dependent, its clearance is an estimate of hepatic blood flow. Following its oral administration, d4-cholate is delivered to the liver via the portal vein. In health (Figure 1A–C), 80% is removed by hepatic uptake in a first pass through the liver—only ∼20% spills over into the systemic circulation, and the measured area under the clearance curve for d4-cholate in peripheral blood is relatively low. With liver disease (Figure 1D–F), diminished hepatocyte uptake and portal venous collaterals increase the spillover, or shunt, of the d4-cholate into the systemic circulation and area under the clearance curve increases.

The OCCT directly measures portal clearance and estimates systemic clearance using a model trained in subjects receiving the dual cholate shunt test, involving intravenous (IV) injection of 13C-cholate and oral d4-cholate administered simultaneously.^38^^,^^39^ The test’s parameters are derived from these clearance values and have been shown to be equivalent to those obtained from the dual (IV and oral) cholate shunt test^40^; have high within-individual reproducibility^41^; and correlate with stage of fibrosis on liver biopsy, likelihood of varices at endoscopy, development of PH, improvement after initiation of drug therapy, and risk for future clinical outcome. Parameters of the test include:

• Disease Severity Index (DSI)—a measure of global liver disease severity on a scale from 0 to 50, with 0 representing the healthiest possible liver function and 50 representing the most severe liver dysfunction. Table 1 summarizes the distribution of DSI values collected from clinical studies and trials across various patient cohorts referenced in this review.

**Table 1:** Summary of Clinical Studies, Clinical Trials, and Real-World Data Sorted by Median (Min–Max) Baseline Disease Severity Index (DSI) and Hepatic Reserve From the Oral Cholate Challenge Test (OCCT) for 1266 Participants

Reference	Study or cohort abbreviation	Description of participants	DSI	Hepatic reserve (%)
McRae et al 2025	CONTROLS-LEAN	26 healthy controls of lean body mass	8.0 (3.9–13.3)	100 (93.2–100)
McRae et al 2025	CONTROLS-OVERWEIGHT	24 healthy controls with body mass index >25 kg/m^2^	12.7 (6.6–16.9)	98.2 (86.8–100)
Helmke et al 2024	PSC-LOW	30 with PSC and low impairment^a^	14.4 (7.5–22.6)	92.6 (70.9–100)
Everson et al 2012	HALT-C-FIBROSIS	168 with fibrotic HCV^a^	15.5 (4.5–33.0)	90.3 (44.3–100)
Harrison et al 2025	HEPION	69 with ≥F3 MASH^a^	16.7 (8.4–33.9)	87.4 (42.0–100)
Kanodia et al 2024	THERAVANCE-CPA	8 with CP A cirrhosis	16.9 (12.1–25.0)	86.2 (66.0–96.7)
Kim et al 2026	FONTAN	50 with Fontan-associated liver disease	17.0 (4.0–29.2)	86.4 (54.0–100)
Alkhouri et al 2024	INTERCEPT	51 with F1-F4 MASH^a^	17.1 (7.7–31.5)	86.5 (47.9–100)
Wieland et al 2021	NIDDK and CU-HVPG	48 with clinically stable chronic liver disease	18.0 (5.2–31.5)	84.7 (47.3–100)
Burton et al 2021	REPRO	32 subjects (16 HCV, 16 MASH)^a^	18.0 (11.3–32.0)	83.0 (46.7–100)
Fallahzedah et al 2021	BAYLOR-COMP	35 with compensated cirrhosis	19.9 (7.2–31.6)	78.6 (47.7–100)
Gordon et al 2025	EAP	129 with chronic liver disease	20.0 (5.5–45.4)	77.8 (11.8–100)
O'Leary et al 2014	SOLAR-1-LTx-FIB	10 liver transplant recipients with fibrosis^a^	20.4 (14–28.2)	77.7 (57.1–93.3)
Helmke et al 2024	PSC-MOD-HIGH	17 with PSC and moderate-to-high impairment^a^	20.6 (15.3–35.5)	75.8 (37.5–88.8)
Alkhouri et al 2025	MADRIGAL	28 with CP A MASH^a^	21.0 (7.7–40.0)	76.3 (26.0–100)
Shiffman et al 2025	SHUNT-V	238 with CP A cirrhosis	21.7 (3.2–41.7)	73.9 (21.7–100)
Everson et al 2012	HALT-C-CIRRHOSIS	109 with cirrhotic HCV^a^	21.9 (7.3–36.7)	73.6 (34.7–100)
Whitaker et al 2026	SMN	89 with cACLD	22.0 (7.5–39.8)	72.8 (26.4–100)
Kanodia et al 2024	THERAVANCE-CPB	7 with CP B cirrhosis	23.6 (13.1–33.5)	67.2 (42.5–97.2)
Lawitz et al 2024	BI	23 with CP A cirrhosis^a^	24.2 (13.1–36.2)	67.9 (35.4–95.6)
O'Leary et al 2014	SOLAR-1-LTx-CIR	11 liver transplant recipients with cirrhosis^a^	27.8 (17.8–37.2)	58.2 (32.9–84.5)
Hoteit et al 2020	HCC	13 with HCC^a^	30.2 (15.3–40.0)	51.5 (31.2–90.1)
Fallahzedah et al 2021	BAYLOR-DECOMP	35 with decompensated cirrhosis	34.0 (11.5–43.7)	41.8 (16.4–100)
Kanodia et al 2024	THERAVANCE-CPC	6 with CP C cirrhosis	36.7 (14.9–41.6)	34.4 (21.4–92.1)
O'Leary et al 2014	SOLAR-1-ESLD	10 with end-stage liver disease^a^	39.1 (29.4–41.7)	28.1 (21.3–52.9)

a For these studies, the DSI and hepatic reserve values at the baseline visit are reported.
• SHUNT%—a measure of portal-systemic shunting, with <24% representing the normal range in healthy lean controls.
• Hepatic reserve—a measure of global liver health similar to DSI but expressed with 100% representing healthy function observed in individuals with lean body mass and 0% representing extreme dysfunction observed in patients with end-stage liver disease (ESLD). Table 1 summarizes the distribution of hepatic reserve values collected from clinical studies and trials across various patient cohorts referenced in this review.

### Real-World Clinical Validation of the Oral Cholate Challenge Test

The OCCT was commercially launched for clinical use in August 2023 with the initiation of an Early Access Program involving 16 clinicians at 5 liver centers across the United States.^42^ The tested patients (n = 129) represented a range of liver disease etiologies and stages. Top clinical uses were (1) informing the decision for endoscopy to test for varices (n = 56, 43%), (2) defining risk for large esophageal varices (LEV) (n = 92, 71%), and (3) monitoring disease progression or treatment effects (n = 33, 26%). The Early Access Program confirmed that DSI ≤18.3 was associated with a low risk of finding esophageal varices on screening esophagogastroduodenoscopy (EGD) and could reduce the need for EGD by 38%, replicating results of a previous study (41%).^43^

In a preliminary real-world analysis of the influence of DSI in clinical decision-making, 87 cases with compensated advanced chronic liver disease (cACLD) were extracted from physician-provided Statements of Medical Necessity (SMN) documenting the reasons for ordering the OCCT.^44^ Among 55 cases analyzed for endoscopy decisions, overall concordance with DSI 18.3 was 93% (96% of cases with DSI ≤18.3 avoided endoscopy, and 90% with DSI >18.3 underwent endoscopy). For 45 cases assessing management intensity, overall concordance was 89% (90% of cases with DSI ≤18.3 had reduced follow-up and 89% with DSI >18.3 had intensified management). Additional studies evaluating the impact of DSI on clinical decision-making are needed to confirm these preliminary findings.

## Specific Applications

### Portal Hypertension and Varices

LEVs pose significant risk yet are often screened for unnecessarily or not at all, particularly in patients with compensated cirrhosis. Current noninvasive strategies, such as vibration-controlled transient elastography (VCTE) and platelet count (PLT),^45^^,^^46^ have limited accuracy in patients with obesity, diabetes, metabolic dysfunction–associated steatotic liver disease (MASLD), or MASH and do not assess liver function directly. The OCCT overcomes these limitations by quantifying hepatic function and portal-systemic shunting, and its DSI correlates strongly with risk for LEV and other lesions of PH, enabling more accurate stratification and reducing unnecessary endoscopy. This section reviews the diagnostic performance of DSI for ruling out LEVs and its broader role in assessing disease severity and guiding endoscopic decisions.

#### Prediction of risk for varices

The first step in leveraging the OCCT to reduce unnecessary endoscopic procedures was defining a DSI cutpoint that could reliably rule out LEV. The Hepatitis C Antiviral Long-Term Treatment against Cirrhosis (HALT-C) trial was a prospective, randomized US multicenter study of low-dose peginterferon monotherapy for active chronic hepatitis C with prespecified EGD and varices endpoints.^14^^,^^47^, ^48^, ^49^ In a substudy of the HALT-C trial, 217 subjects underwent both HepQuant testing and EGD. The cutpoint of DSI 18.3 had a sensitivity of >95% for endoscopically confirmed LEV.^43^

The cutpoint of DSI 18.3 was subsequently validated in a prospective, blinded, multicenter US pivotal study, SHUNT-V, in which 238 subjects with compensated Child-Pugh (CP) A cirrhosis, inclusive of all etiologies of liver disease, underwent both HepQuant testing and an EGD.^50^ The mean body mass index was 33.4 kg/m^2^, and 52.1% had MASLD/MASH as the underlying etiology of cirrhosis. DSI >18.3 predicted the presence of LEV in 26 of 27 cases defined by EGD (sensitivity 96%). Applying a DSI of ≤18.3 cutoff for LEV, 27% of EGDs would have been avoided. In the combined data from HALT-C and SHUNT-V, applying DSI ≤18.3 would have avoided 41% of EGDs. In addition, a higher cutpoint of DSI ≤24 with a second criterion of PLT >135,000 nL^−1^ would have avoided 57% of EGDs.^51^ Furthermore, these cutpoints have correlated with risk for clinical outcome and can aid decisions regarding repeat EGD and time interval to clinical follow-up in patients with no or small varices.^52^

The DSI cutoff 18.3 also demonstrated excellent diagnostic performance at capturing all clinically significant lesions of PH, including gastric varices and severe portal hypertensive gastropathy.^50^ These findings illuminate the potential for the OCCT to transform clinical practice by enabling clinicians to safely avoid unnecessary endoscopies while improving patient care and resource utilization.

#### Comparison with standard laboratory values and other noninvasive tests

**Figure 2:**
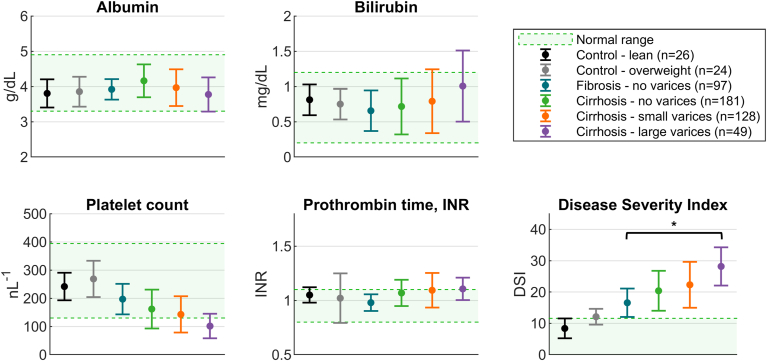
In an analysis of 455 patients with advanced fibrosis or compensated cirrhosis and 50 healthy controls,^43^ values for standard “liver function tests” (total bilirubin, albumin, and international normalized ratio [INR]) overlapped completely with the normal range, whereas the Disease Severity Index (DSI) from the oral cholate challenge test distinguished liver patients from healthy controls (∗) and demonstrated stepwise worsening from no varices to small or large varices.^53^ Values represent mean ± 1 SD. SD, standard deviation.

Comparing the OCCT to conventional laboratory tests and other noninvasive measures highlights its unique ability to distinguish liver disease severity and PH risk. In an analysis of 455 patients with advanced fibrosis or compensated cirrhosis, values for total bilirubin, albumin, and international normalized ratio (INR) overlapped completely with the values for healthy controls, whereas all cholate challenge parameters, including DSI, distinguished liver patients from healthy controls and demonstrated stepwise worsening from no varices to small or large varices (Figure 2).^43^

**Figure 3:**
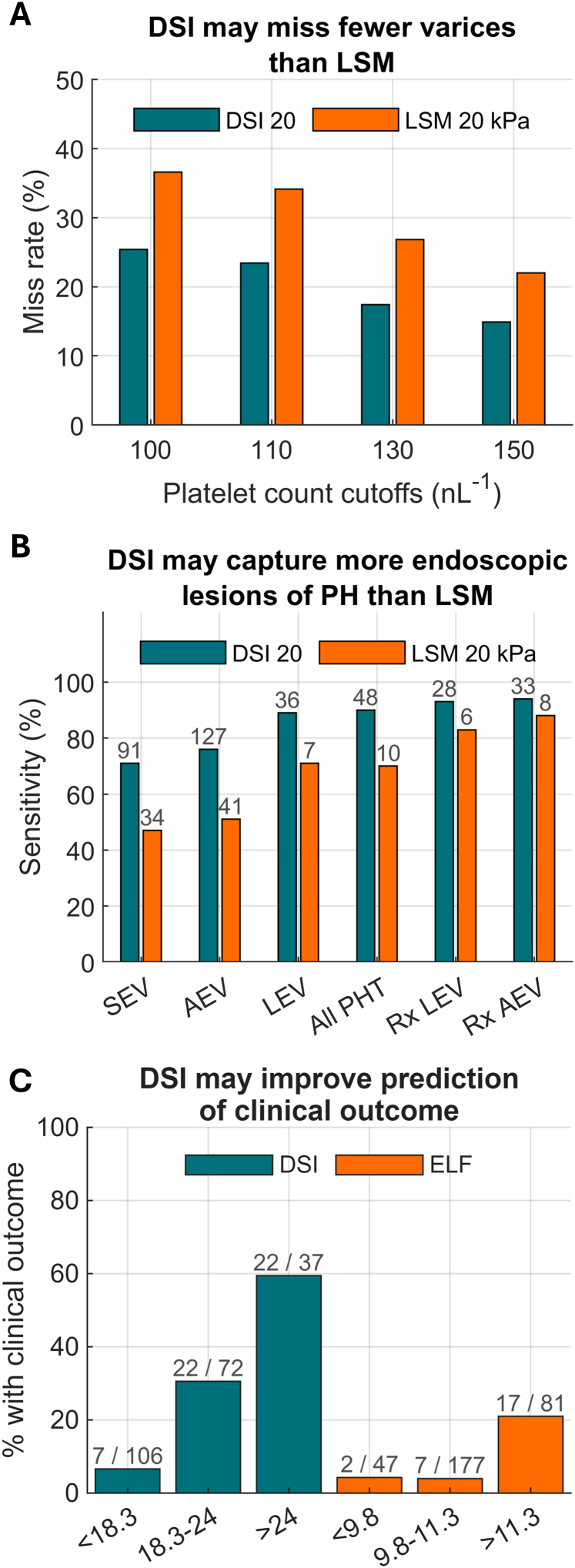
Comparison of DSI to other noninvasive tests. DSI from the OCCT (n = 275) compared to liver stiffness measurements (LSM, n = 86) for miss rate for any esophageal varices at various platelet count cutoffs used in the Baveno criteria (A) and for diagnostic sensitivity in detecting endoscopic lesions of PH (B). Prediction of risk for decompensation was evaluated by DSI in patients with cACLD due to chronic hepatitis C (n = 215) and compared to the risk reported in the U.S. Food and Drug Administration De Novo approval for ELF in MASLD (n = 305) (C). AEV, any esophageal varices; LEVs, large esophageal varices; Rx AEV, treated any esophageal varices; Rx LEV, banded at EGD or varices medication prescribed post-EGD; SEV, small esophageal varices; All PHT, all significant endoscopic lesions of PH (LEV, Rx LEV, Rx AEV, large gastric varices, varices with red wale signs, and severe portal hypertensive gastropathy).

Although DSI correlates with liver stiffness measurements (LSM) by VCTE, the correlation is relatively weak.^54^ In the SHUNT-V study of 275 subjects with cACLD, only 86 subjects had VCTE LSM.^42^^,^^43^ A post hoc analysis compared the diagnostic performance of DSI 20 vs LSM 20 kPa, since these values had equivalent rates of endoscopy avoidance to check for varices (43%). DSI detected more of the varices of any size compared with LSM for any given PLT cutoff used in the various Baveno criteria (Figure 3A). Similarly, DSI 20 captured more endoscopic lesions of PH than LSM (ie, higher sensitivity for any varices, varices needing treatment, large gastric varices, varices with red wale signs, and severe portal hypertensive gastropathy) (Figure 3B).

DSI has also been shown to correlate with enhanced liver fibrosis (ELF) score—although weakly.^54^ The performance in predicting risk for decompensation in patients with compensated MASH cirrhosis (n = 305) was 4.3% for ELF <9.8, 4.0% for ELF 9.8% to 11.3%, and 21.0% for ELF >11.3 (Figure 3C) over a median of about 15 months of follow-up.^55^ In a different study population, the performance of DSI in predicting risk for decompensation in patients with cACLD (n = 215) was 6.6% for DSI <18.3, 30.6% for DSI 18.3% to 24%, and 59.5% for DSI >24 over an average of 5.3 years of follow-up.^56^

These comparisons to standard labs, LSM, and ELF suggest that DSI may offer some advantages in estimating the risk of decompensation. The relatively weak correlations with LSM and ELF, despite strong association with varices and clinical outcomes, suggest that DSI may add unique information about the patient not otherwise appreciated by other noninvasive tests.

#### Current status of noninvasive tests as surrogates for hepatic venous pressure gradient

PH drives major complications in cACLD, including ascites, variceal bleeding, and encephalopathy.^46^ Clinically significant PH (CSPH) is defined by a hepatic venous pressure gradient (HVPG) ≥10 mmHg, the point at or above which LEV may be identified.^57^ Treatment with either nonselective beta-blockers (NSBBs) or esophageal variceal ligation may be initiated in these patients to reduce variceal hemorrhage, hepatic decompensation, morbidity, and mortality.^58^ Although HVPG is the gold standard, it is invasive, costly, technically demanding, and unreliable in the presence of veno-venous collaterals,^50^ while endoscopic approaches carry higher procedural risks in cirrhosis.^59^

**Figure 4:**
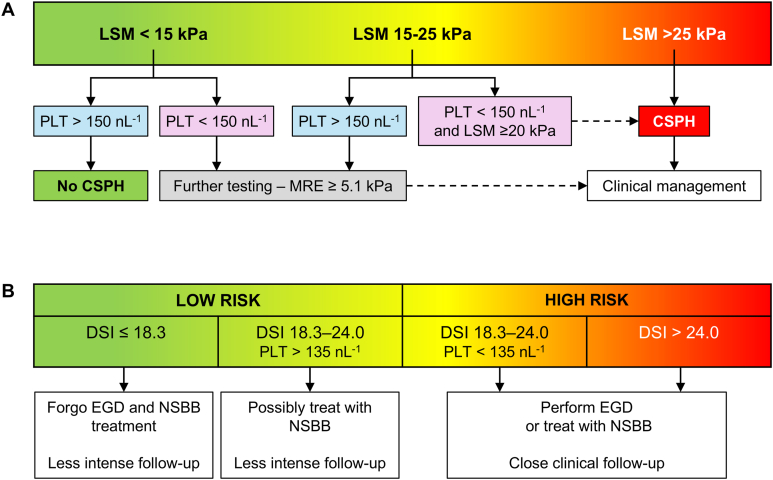
Guidelines proposed by AASLD (A) and proposed algorithm with DSI (B) for detection and clinical management of patients at risk for clinically significant portal hypertension (CSPH). The boundaries for the DSI cutpoints were established in studies linking DSI to risk for large esophageal varices^43^^,^^50^^,^^51^ and are compared to the guideline proposed by AASLD.^67^ The addition of platelet count to DSI for the DSI range 18.3–24 clarifies risk of CSPH and large varices, which could aid the determination of these risks within the LSM range from 15 to 25 kPa. AASLD, American Association for the Study of Liver Diseases; MRE, magnetic resonance elastography.

In compensated cirrhosis, guidelines support proceeding with a screening EGD to detect and treat LEV^60^, ^61^, ^62^, ^63^, ^64^, ^65^, ^66^ or instituting empiric treatment with NSBBs in patients with kPa ≥25 by VCTE (Figure 4).^67^^,^^68^ However, the current algorithm may not be as effective in obesity, and use of EGD without risk stratification yields LEV in only 10%–15% of cases. Although the yield of LEV is increased by prescreening with the combination of PLT and VCTE LSM, LSM measurements may be influenced by the underlying etiology and stage of liver disease (especially in patients with advanced MASH), and less accurate in patients with obesity, diabetes, MASH, and older age.^46^^,^^69^^,^^70^ Many other pitfalls of LSM have been noted, including wide intraindividual and interobserver variance.^71^

**Figure 5:**
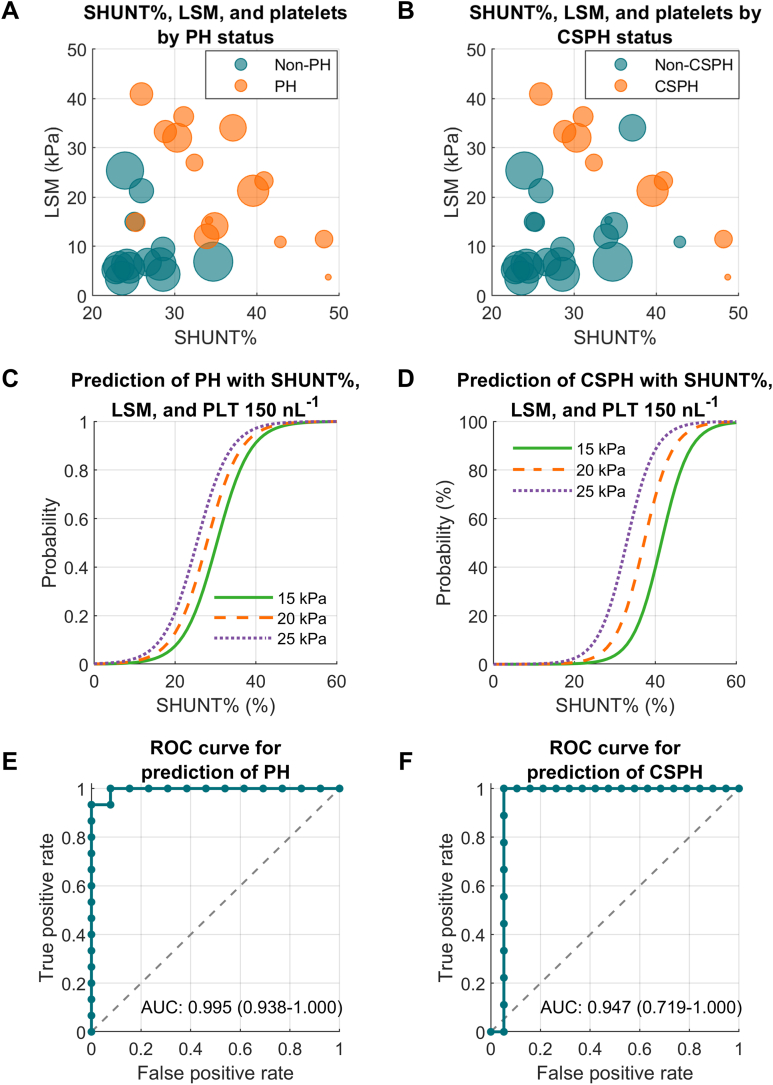
Prediction of portal hypertension (PH) and clinically significant PH (CSPH) by SHUNT%, liver stiffness measurement (LSM), and platelet count (PLT). Bubble charts show the distribution of SHUNT% and LSM by PH (A) or CSPH (B) status, with bubble size indicating PLT (minimum 72 nL^−1^, maximum 279 nL^−1^). Logistic regression plots show the probability of PH (C) or CSPH (D) for a given SHUNT% at 3 levels of LSM (15, 20, and 25 kPa) and one level of platelet count (150 nL^−1^). Receiver-operating characteristic (ROC) curves show the diagnostic performance of the multivariable logistic regression models including SHUNT%, LSM, and PLT for the prediction of PH (E), and CSPH (F). AUC, area under the curve.

OCCT parameters address these limitations: SHUNT% strongly predicts PH and CSPH (AUROCs 0.92 and 0.86) across cohorts undergoing HVPG or direct portal pressure measurement.^72^ In the subset with chronic hepatitis C, models combining SHUNT%, LSM, and platelets achieved near-perfect accuracy with AUROCs of 0.995 for PH and 0.947 for CSPH (Figure 5).^32^^,^^72^^,^^73^ These findings indicate that DSI and SHUNT% may serve as robust noninvasive surrogates for PH and CSPH, supporting their comparison with American Association for the Study of Liver Diseases CSPH criteria (Figure 4).^67^ Additional external validation of these models is warranted.

#### Cost-effectiveness

Management decisions based on DSI—such as avoiding unnecessary endoscopies, focusing care on higher-risk patients, and reducing monitoring in low-risk groups—were shown to be cost-effective in a Markov decision tree modeled on a base population of 100,000 patients with chronic liver disease.^74^ The model used published data on epidemiology, costs, and quality of life to compare the OCCT with the standard of care. The model evaluated the impact of DSI-guided decisions on the performance of endoscopy, reduced allocation of resources in low-risk patients, reduction in decompensation in high-risk patients, hospitalizations, QALYs, and mortality. Sensitivity analysis showed that 8 key factors accounted for most of the uncertainty in determining when the strategy became cost-effective, including progression rates, costs of managing cirrhosis, and the proportion of patients with DSI >18.3. At a test price of $3,250, using the OCCT was estimated to save 2740 lives over 5 years and became cost-saving within 2 years at prices ≤$3,213 and within 5 years at ≤$4,100. Overall, the model demonstrated meaningful economic and clinical benefit for managing patients with cACLD at risk for LEV.

### Predicting Clinical Outcomes

Across several studies, DSI has been shown to predict decompensation with better performance characteristics than CP score, MELD (Model for End-Stage Liver Disease), and MELD-Na (sodium MELD) scores.^43^^,^^75^ This section highlights the prediction of clinical outcome using (1) baseline DSI and SHUNT% and (2) change in DSI from baseline at follow-up.

#### Prediction of clinical outcome using baseline Disease Severity Index and SHUNT%

##### Chronic hepatitis C

In the HALT-C trial's Quantitative Liver Function Test (QLFT) ancillary study, cACLD patients with active chronic hepatitis C and advanced fibrosis or compensated cirrhosis were treated with maintenance interferon and followed for an average of 5.3 years.^14^^,^^56^ In the subset with cirrhosis, the likelihood of clinical outcome was 13%, 30%, and 68% for baseline DSI ≤18.3, 18.3 to 24, and greater than 24, respectively. In the subset with noncirrhotic fibrosis, the likelihood of clinical outcome was 5%, 23%, and 38% for the same ranges of DSI. This suggests that a patient classified as having noncirrhotic fibrosis with DSI >24 is at greater risk for clinical outcome than the patient classified as having cirrhosis but with DSI <24. Mean baseline DSI correlated with the effectiveness of interferon/ribavirin treatment. The DSI was 16.5 in subjects achieving sustained virologic response [SVR], 18.1 in subjects with partial response who had breakthrough or relapse [BT/R], and 19.1 in subjects with nonresponse [NR], implying that the degree of functional impairment at baseline may predict subsequent treatment response.

##### Compensated and decompensated liver disease

In a long-term follow-up study (average 4.2 years),^76^ of 70 patients with cirrhosis (35 compensated and 35 decompensated/recompensated), baseline DSI was significantly higher in the decompensated group (32.6 vs 20.9, *P* < .001). Thirty-four percent of compensated patients experienced decompensation, liver transplantation, or liver-related death; 46% decompensated patients underwent transplantation or died. DSI ≥24 independently predicted liver transplantation or liver-related death (*P* = .02), and patients above this threshold had significantly higher cumulative event rates (*P* = .005). Among compensated patients, DSI (AUROC 0.74) outperformed MELD-Na (AUROC 0.62), and combining MELD-Na with DSI did not improve accuracy.

##### Primary sclerosing cholangitis

The relationship of DSI and SHUNT% to clinical outcome was evaluated prospectively in a study of 47 patients with primary sclerosing cholangitis (PSC) (mean follow-up of 391 days).^77^ Clinical outcomes occurring after the baseline test were identified from patient histories and chart reviews. Baseline DSI was significantly higher in patients who had findings consistent with PH at enrollment (n = 23) or who had a prior history of hepatic decompensation (n = 8) relative to those with no findings or prior history (both *P* < .002). DSI was a strong baseline predictor of clinical outcome (AUROC 0.83) and was significantly higher in the 13 subjects who experienced clinical outcome compared to those who were clinically stable (24.6 vs 16.5, *P* < .001). In univariate analyses of a variety of noninvasive tests or clinical algorithms, SHUNT% and DSI were the strongest predictors of clinical outcome.

##### Fontan-associated liver disease

The relationship of DSI and SHUNT% to clinical outcome was evaluated in 2 studies of Fontan-associated liver disease (FALD) encompassing 50 adults with Fontan circulation.^78^, ^79^, ^80^ Twenty-three subjects (46%) had SHUNT% >30%, a cutpoint associated with greater risk for clinical outcome.^56^ Transplant-free survival in adults with Fontan circulation was strongly associated with SHUNT%, with each 10% increase in SHUNT% conferring a 1.6-fold higher hazard of death or transplant. Elevated SHUNT% also conferred a significantly higher odds of a composite adverse outcome (death, transplant, new onset heart failure, ascites, protein-losing enteropathy, or HCC), whereas the Varices, Ascites, Splenomegaly, Thrombocytopenia score did not demonstrate prognostic value in this cohort. Patients with SHUNT% >30% had significantly worse 5-year outcomes compared to those with SHUNT% ≤30% (60% vs 88%). Only one of the 34 (3%) patients with SHUNT% ≤30% experienced the primary outcome event (death or transplant) within 4 years of the study, whereas 4 of 14 (29%) patients with SHUNT% >30% did. These findings support SHUNT% as a functional biomarker that captures key hemodynamic and hepatic derangements in FALD and may aid in optimizing timing of transplant referral.

#### Prediction of clinical outcome using change in Disease Severity Index

##### Natural progression of chronic hepatitis C

In a longitudinal study of 215 patients with chronic hepatitis C,^14^ the probability of clinical outcome was modeled using baseline DSI and its rate of change over time (Risk of Adverse Clinical Events [RISK ACE] model).^56^ DSI was measured at baseline and then subsequently at approximately 24 months. Patients were followed for an average of 5.3 years for clinical events including death, a sustained 2-point increase in CP score, variceal hemorrhage, ascites, and encephalopathy. Fifty patients experienced decompensation, and both higher baseline DSI and more rapid increases in DSI were strongly associated with these outcomes (*P* < .001). Multivariable analysis confirmed baseline DSI (HR 1.2, *P* < .0001) and rate of change (HR 16.9, *P* = .0008) as significant predictors, whereas Ishak fibrosis stage was not. These findings suggest that DSI, and rate of change in DSI, may be more sensitive than fibrosis staging at predicting risk for future clinical events.

It is important to note that the RISK ACE models were developed from a cohort of active chronic hepatitis C patients, where both reduction in DSI and stability of DSI were associated with lower risk for clinical outcome.^56^ Although the main etiology of liver disease in this study population was chronic hepatitis C, the high prevalence of overweight body habitus, obesity, and alcohol use suggests that the Cox models could be applicable to both MASLD and ALD.

##### Natural progression of primary sclerosing cholangitis

Further supportive evidence comes from a study of patients with PSC.^77^ In this cohort, 40 patients were followed for approximately 1 year, 10 experienced a clinical outcome, while 30 remained stable. Baseline and follow-up DSI were significantly higher in those with outcomes (*P* = .004), and DSI increased significantly between baseline and follow-up visits in this group compared with stable patients (ΔDSI of +2.3 vs −0.4, *P* = .042). These findings suggest that rising DSI over time reflects worsening in hepatic function and may serve as an early signal of clinical deterioration in PSC.

##### Antiviral therapy in patients with chronic hepatitis C

The changes in DSI between baseline (n = 217) and a second DSI at approximately 2 years (n = 171) were modeled for estimating risk for clinical outcome (RISK ACE model from the QLFT ancillary study).^56^ Changes between the estimated clinical risk at baseline and the estimated risk as a result of treatment were compared between SVR, partial responders experiencing BT/R, and NR (HepQuant internal data). The changes in risk aligned with treatment response: a 38% reduction in the SVR group, 16% reduction in BT/R, and a 10% increase in nonresponders. Furthermore, the estimated risk of clinical outcome after treatment in the QLFT substudy was compared to observed rates of clinical outcomes reported from the main trial over 7 years.^81^ Estimated risk for clinical outcome from RISK ACE closely mirrored the observed risk for clinical outcome: SVR, estimated 7.8% vs observed 3.6%; BT/R, estimated 15.7% vs observed 10.4%; and, NR, estimated 22.7% vs observed 25.2%. These findings highlight the potential utility of dynamic changes in DSI for predicting long-term outcomes and differentiating treatment response categories.

##### Resmetirom treatment of metabolic dysfunction–associated steatohepatitis cirrhosis

This section describes the use of DSI measurements in the RISK ACE model to estimate the risk for clinical outcome in subjects treated with resmetirom. Refer to the Monitoring Treatment Effect section below for additional results of this study.

In the MAESTRO-NAFLD-1 substudy, 23 subjects with compensated MASH cirrhosis were treated with resmetirom and had DSI measured at baseline, week 28, and week 48.^54^ The DSI measurements were then used to estimate the risk for clinical outcome (RISK ACE). The predicted risk of clinical outcome decreased from 11%, based on the baseline DSI, to 7%, based on the change in DSI after 48 weeks of resmetirom (*P* = .041). This estimated risk generated in the substudy of 23 subjects closely mirrored the observed 2-year outcome rate in the larger MAESTRO-NAFLD-1 cohort of 122 subjects (6%).^82^ The greatest improvement occurred in patients with the highest baseline DSI, highlighting the potential of DSI to quantify treatment benefit and project long-term risk reduction in MASH. The estimated decline in clinical risk from 11% to 7% yielded a relative risk reduction of 36% which was nearly identical to the estimated risk reduction of 38% from SVR in chronic hepatitis C virus. This suggests that DSI and RISK ACE may be useful in comparing the efficacy of interventions across various etiologies of liver disease and diverse types of treatments.

### Monitoring Treatment Effect

The therapeutic drug monitoring performance of the OCCT and DSI is driven by 2 key attributes. First, DSI provides a dynamic, real-time assessment of liver function and physiology, capturing changes as they occur—an advantage in defining effects early during treatment. This attribute has been defined across all liver disease etiologies and stages of disease and across a broad range of clinical studies. Second, DSI has excellent reproducibility^41^ whereby a 2-unit change in DSI represents reliable functional response to therapy (ie, >2-point decrease are responders, within ±2 points are stable responders, and >2-point increase are nonresponders).^54^^,^^83^^,^^84^ The sections below summarize a subset of the many drug trials in which DSI was used to detect treatment effects (Table 2).

**Table 2:** Summary of Drug Trials Demonstrating Treatment Effects Detected by Change in DSI

Trial/design	Population	Intervention	Duration	Outcome of study
INTERCEPT-117 (Intercept Pharmaceuticals): Phase 1, randomized, placebo-controlled, double-blind trial^85^	MASH F1–F4 (n = 51)	Obeticholic acid (10 mg, 25 mg)	85 d	Dose-dependent response and significant treatment effect in the OCA 25 mg arm detected by change in DSI
BI 685509 (Boehringer Ingelheim): Phase 1b, randomized, placebo-controlled, double-blind trial^84^	Child-Pugh A cirrhosis (n = 24)	Avenciguat (1 mg BID, 2 mg BID, 3 mg BID)	27 d	Dose-dependent improvement in DSI and treatment effect in the 3 mg arm (*P* < .10)
ALTITUDE-NASH (Hepion Pharmaceuticals): Phase 2, randomized, open-label trial^83^	MASH ≥ F3 (n = 70)	Rencofilstat (75 mg, 150 mg, 225 mg)	120 d	Significant reductions in DSI detected at 60 and 120 d in the 225 mg arm
SOLAR-1 sub-study (Gilead Sciences)^86^	Liver transplant recipients with advanced hepatitis C (n = 29)	Ledipasvir/sofosbuvir and ribavirin	48 wk	DSI showed a significant reduction in non-cirrhotic patients, non-significant reduction in patients with cirrhosis, and remained unchanged in patients with end-stage liver disease
MAESTRO-NAFLD-1 OLE (Madrigal Pharmaceuticals): Phase 3, open-label trial^54^	Compensated MASH cirrhosis (n = 28)	Resmetirom 80 mg	48 wk	Significant reduction in the estimated clinical risk by Cox model with DSI and change in DSI

#### Early functional improvement in fibrotic metabolic dysfunction–associated steatohepatitis during treatment with obeticholic acid

**Figure 6:**
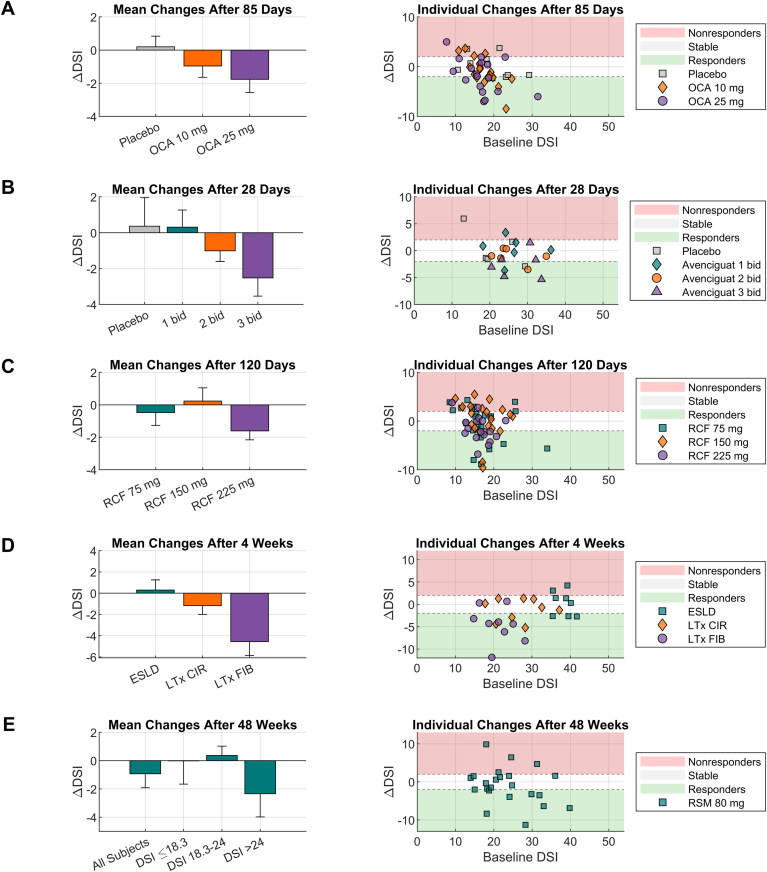
Disease Severity Index (DSI) from the OCCT detected treatment responses in several drug trials, including: (A) obeticholic acid (OCA) in patients with fibrotic stage MASH, (B) avenciguat treatment in patients with Child-Pugh A cirrhosis, (C) rencofilstat treatment in patients with advanced fibrosis MASH, (D) ledipasvir/sofosbuvir in liver transplant recipients and patients with advanced cirrhosis, (E) resmetirom treatment in patients with MASH-related Child-Pugh A cirrhosis. LTx CIR, liver transplant recipients with cirrhosis; LTx FIB, liver transplant recipients with fibrosis; RCF, rencofilstat.

Obeticholic acid (OCA) demonstrated antifibrotic efficacy in patients with F2–F3 MASH in the phase 3 REGENERATE (Randomized Global Phase 3 Study to Evaluate the Impact on NASH With Fibrosis of Obeticholic Acid Treatment) trial.^87^ In a parallel double-blind, placebo-controlled phase 1 study,^85^ the OCCT was used to assess hepatic effects across the fibrosis spectrum (F1–F4). Fifty-one adults with biopsy-confirmed MASH were randomized to placebo (n = 11), OCA 10 mg (n = 20), or OCA 25 mg (n = 20) daily for 85 days. The primary endpoint was the change in DSI from baseline to day 85. Mean DSI changes were +0.2 for placebo, −1.0 for OCA 10 mg, and −1.8 for OCA 25 mg (*P* = .04), and there was a higher proportion of responders (≥2-point reduction in DSI) in the OCA groups compared with placebo (Figure 6). These findings support DSI as a sensitive early indicator of dose-dependent therapeutic response.

In September 2025, Intercept Pharmaceuticals announced their voluntary withdrawal of OCA from the U.S. market due to safety concerns at the request of the U.S. Food and Drug Administration.^88^ Despite these recent developments, the original trial observed reductions in liver markers (alanine aminotransferase [ALT], aspartate aminotransferase [AST], gamma-glutamyl transferase [GGT]) between baseline and day 85 that were consistent with the findings with DSI. There were no serious adverse events during the study, and only 1 hepatic adverse event was reported (an F3 subject in the OCA 25 mg group had an asymptomatic increase in alkaline phosphatase).

#### Hepatic improvement within 27 days of avenciguat treatment in Child-Pugh A cirrhosis

Soluble guanylyl cyclase activators lower portal pressure and improve hepatic perfusion, offering a potential alternative to NSBBs. Avenciguat, a nitric oxide–independent soluble guanylyl cyclase activator, has shown beneficial effects in preclinical models. Its hepatic impact was evaluated using the OCCT in a phase 1b, double-blind, placebo-controlled, dose-escalation study.^84^^,^^89^ Patients with CP A cirrhosis received avenciguat at 1 mg, 2 mg, or 3 mg twice daily (n = 6 per group) or placebo (n = 5) for 27 days. Avenciguat produced dose-dependent reductions in DSI: +0.4 for placebo, +0.3 for 1 mg, −1.0 for 2 mg, and −2.5 for 3 mg (Figure 6B). These findings demonstrate that the OCCT can detect early, dose-dependent improvements in hepatic function within 4 weeks of treatment, highlighting its utility as a sensitive early indicator of treatment efficacy.

#### Improvement in liver function with rencofilstat treatment in metabolic dysfunction–associated steatohepatitis with advanced fibrosis

Rencofilstat, a cyclophilin inhibitor, targets hepatic inflammation and fibrosis, potentially improving liver function and reducing portal-systemic shunting. Its effect was evaluated using the OCCT in a phase 2 open-label study of 70 patients with biopsy- or AGILE3-confirmed ≥ F3 MASH.^83^ Study participants and investigators were blinded to treatment arms, and subjects were randomized to rencofilstat 75 mg/d (n = 24), 150 mg/d (n = 23), or 225 mg/d (n = 23). DSI was measured at baseline, day 60, and day 120. The 225 mg/d group showed significant DSI reductions at day 60 (−1.3, *P* = .02) and day 120 (−1.6, *P* = .02), with 56% classified as responders by day 120 (*P* = .05) (Figure 6C). Across all arms, the greatest improvement occurred in patients with baseline DSI >18.3 (ΔDSI −2.6, *P* = .005). These findings indicate that rencofilstat at 225 mg/d enhances hepatic function and supports DSI as a sensitive marker for short-term treatment effects.

#### Early hepatic improvement with ledipasvir/sofosbuvir in liver transplant recipients and patients with advanced cirrhosis

In the SOLAR-1 trial,^86^ liver transplant (LTx) recipients with fibrosis or cirrhosis and patients with ESLD awaiting transplantation received ledipasvir/sofosbuvir plus ribavirin. DSI was assessed at baseline and at weeks 4, 24, 36, and 48 in 3 groups: noncirrhotic (n = 9), cirrhotic (n = 9), and ESLD (n = 8). After 4 weeks, DSI declined significantly in the noncirrhotic group (ΔDSI −4.6, *P* < .01), showed a nonsignificant reduction in the cirrhotic group (ΔDSI −1.2, *P* = .20), and remained unchanged in ESLD patients (ΔDSI +0.3, *P* = .77) (Figure 6D). Throughout the 48 weeks of testing, DSI remained significantly reduced in noncirrhotic LTx recipients, trended downward by week 24 but was not sustained in cirrhotic LTx recipients, and did not change in ESLD patients. The favorable early improvements in function in noncirrhotic patients imply a more dynamic reversibility of the changes to the hepatic microcirculation with resolution of the necroinflammation. Slower improvement or lack of early improvement in function in the advanced disease subjects suggests that longer follow-up may be required to demonstrate recovery. These findings underscore DSI’s sensitivity in detecting early functional responses to antiviral therapy across a spectrum of stages of fibrosis and clinical severity.

#### Improvement in response to resmetirom treatment in metabolic dysfunction–associated steatohepatitis–related Child-Pugh A cirrhosis

Resmetirom, a liver-targeted thyroid hormone receptor-β agonist, is approved for MASH with moderate to advanced fibrosis (F2–F3) and currently under investigation for cirrhotic MASH. In the MAESTRO-NAFLD-1 phase 3 trial,^54^ patients with well-compensated (CP A) MASH cirrhosis received open-label resmetirom 80 mg daily for 52 weeks. DSI was measured at baseline (n = 28), week 28 (n = 23), and week 48 (n = 23). Although mean reductions at weeks 28 and 48 were not statistically significant, 83% of participants demonstrated either stability or improvement by week 48 (Figure 6E). Responders increased from 14% at week 28, to 38% at week 48 (*P* = .03), with greater improvement in those with baseline DSI >18.3. These findings align with resmetirom’s mechanism, rapid reduction of intrahepatic triglycerides and inflammation followed by fibrosis remodeling, suggesting potential for further functional improvement measured by DSI over time.

#### Preservation of Disease Severity Index and reduced SHUNT% by statins

In the SHUNT-V study, we evaluated the association of maintenance drug therapy with the degree of hepatic dysfunction and portal-systemic shunting.^90^ In univariable regression, metformin and statins were associated with lower DSI and SHUNT%. In multivariable regression, lower DSI was attributable to statins (*P* = .035) and metformin (*P* = .056). The combined use of lipid-lowering and antidiabetic drugs, compared to no use, was associated with a 19% reduction in DSI. These results suggest that concomitant use of statins alone or in combination with metformin was independently associated with preserved hepatic function (DSI) and reduced portal-systemic shunting (SHUNT%).

## Other Applications and Special Populations

The following sections highlight interesting applications and special populations for which additional data with the OCCT is warranted.

### Fontan-Associated Liver Disease

In a cohort of 35 adults with FALD,^79^ the prototypical HepQuant SHUNT test showed reduced portal clearance and increased SHUNT%, indicating impaired liver function.^91^ Higher Fontan pressures correlated with reduced portal inflow and compensatory arterial inflow, highlighting its potential for grading FALD severity and monitoring progression. And, as noted above, a single SHUNT% >30%, generated from the HepQuant SHUNT test, may predict risk for clinical outcome over a 5-year period of follow-up.^78^ Additional studies across multiple cohorts will be needed to further validate the predictive value of SHUNT% and DSI in FALD.

### Hepatocellular Carcinoma

In a pilot study of patients with HCC undergoing liver-directed therapy (LDT), the OCCT detected meaningful declines in liver reserve that were not captured by MELD or CP scores.^92^ LDT was associated with reduced portal cholate clearance and worsening DSI, while standard laboratory scores remained largely unchanged. Baseline DSI also stratified risk for hepatotoxicity: 60% of CP B patients with DSI >35 experienced toxicity, compared with none below 35. These findings highlight DSI as a sensitive physiologic marker that better reflects hepatic vulnerability, identifies subclinical functional decline after LDT, and improves risk stratification in patients with HCC.

### Extracorporeal Liver Circuits

Cholate challenge testing by IV route proved highly informative in a study of extracorporeal liver cross-circulation (ELC) in brain-dead decedents, enabling simultaneous quantification of hepatic filtration rates for both native human and genetically modified porcine livers (EGEN-5784).^93^ We observed that initiation of ELC consistently produced substantial increases in total hepatic filtration rate, often exceeding the upper limit of the healthy reference range (5.4 ± 1.0 mL/min/kg). Across 4 decedents, the combined clearance capacity of the native human and EGEN-5784 livers augmented global hepatic function by 47%–115% compared to pre-ELC values, with both organs maintaining stable performance over prolonged support periods of up to 102 hours. ELC may address an unmet need for temporary liver support in patients with acute or acute-on-chronic liver failure. Tracking ELC and native liver functions during this critical period may aid decisions regarding withdrawal or continuation of ELC and whether to consider liver transplantation.

## Future Applications

Future applications for which more data are needed include:

• Polycystic liver disease
• Pediatric liver diseases
• Drug-induced liver injury
• Noncirrhotic PH
• Bariatric surgery
• Portal-sinusoidal vascular disorders
• ALD including alcohol-associated hepatitis
• Liver transplantation
  ○ Living donor selection of recipients
  ○ Evaluation of living donors
  ○ Simultaneous liver-kidney transplantation versus kidney transplant alone
  ○ Post-transplant graft function
• Assessments of cACLD patients for surgical procedures
• ELC

## Summary

In this review, we provide a brief history of achievements and pitfalls in the quantification of liver function and physiology and have highlighted the current and ongoing results with the OCCT. Studies supporting the OCCT as a valid measurement of liver function and physiology have included analytical studies of reproducibility, precision, accuracy, and lack of interference by both endogenous and exogenous compounds, and clinical validation from studies encompassing the broad spectrum of stages and etiologies of chronic liver disease. The results from these studies, summarized in Table 1, warrant further use and evaluation of the OCCT in both the clinic and clinical trials. Referring to Table 1, the cutpoint of DSI 18.3 indicates risk for LEV, corresponding to the transition to CSPH. The cutpoint of DSI 24 corresponds to transition from clinical compensation to clinical decompensation. The corresponding cutpoints for hepatic reserve are approximately 80% and 60%. Further refinement and validation of these cutpoints will be a goal for future studies. Nonetheless, the current emerging results equating functional and physiologic impairment quantified by the OCCT show consistency across a wide range of hepatic conditions.

## Conclusion

The OCCT introduces a transformative approach to liver disease management by providing a simple, noninvasive, and physiologically grounded measure of hepatic function and portal-systemic shunting. Unlike conventional tests, it delivers dynamic, quantitative insights that correlate with disease severity, risk for PH, and clinical outcomes. Its validated performance in guiding endoscopy decisions, predicting prognosis, and monitoring therapeutic response positions the OCCT as a practical tool for precision hepatology. By enabling earlier intervention and more personalized care, this test is poised to redefine the assessment of the patient with chronic liver disease and accelerate drug development in chronic liver disease.
